# Induction of Viable but Nonculturable *Escherichia coli* O157:H7 by High Pressure CO_2_ and Its Characteristics

**DOI:** 10.1371/journal.pone.0062388

**Published:** 2013-04-23

**Authors:** Feng Zhao, Xiufang Bi, Yanling Hao, Xiaojun Liao

**Affiliations:** 1 College of Food Science and Nutritional Engineering, China Agricultural University, Beijing, China; 2 Chinese National Engineering Research Centre for Fruit and Vegetable Processing, Beijing, China; 3 Key Lab of Fruit and Vegetable Processing, Ministry of Agriculture, Beijing, China; Louisiana State University and A & M College, United States of America

## Abstract

The viable but nonculturable (VBNC) state is a survival strategy adopted by many pathogens when exposed to harsh environmental stresses. In this study, we investigated for the first time that whether high pressure CO_2_ (HPCD), one of the nonthermal pasteurization techniques, can induce *Escherichia coli* O157:H7 into the VBNC state. By measuring plate counts, viable cell counts and total cell counts, *E. coli* O157:H7 in 0.85% NaCl solution (pH 7.0) was able to enter the VBNC state by HPCD treatment at 5 MPa and four temperatures (25°C, 31°C, 34°C and 37°C). Meanwhile, with the improvement of treatment temperature, the time required for *E. coli* O157:H7 to enter VBNC state would shorten. Enzymatic activities in these VBNC cells were lower than those in the exponential-phase cells by using API ZYM kit, which were also reduced with increasing the treatment temperature, but the mechanical resistance of the VBNC cells to sonication was enhanced. These results further confirmed VBNC state was a self-protection mechanism for some bacteria, which minimized cellular energetic requirements and increased the cell resistance. When incubated in tryptic soy broth at 37°C, the VBNC cells induced by HPCD treatment at 25°C, 31°C and 34°C achieved resuscitation, but their resuscitation capabilities decreased with increasing the treatment temperature. Furthermore, electron microscopy revealed changes in the morphology and interior structure of the VBNC cells and the resuscitated cells. These results demonstrated that HPCD could induce *E. coli* O157:H7 into the VBNC state. Therefore, it is necessary to detect if there exist VBNC microorganisms in HPCD-treated products by molecular-based methods for food safety.

## Introduction

*Escherichia coli* O157:H7, which was first recognized as a human pathogen in 1982 [1], can cause haemorrhagic colitis and haemolytic uremic syndrome (HUS) in humans [2]. This pathogen has been implicated in large food-borne outbreaks all over the world, such as Canada, UK, Japan and Scotland [3]. The majority of outbreaks caused by *E. coli* O157:H7 have been linked to the consumption of contaminated foods and drinking water [4], [5]. In 2011, the disease outbreaks due to *E. coli* O157:H7 contamination of spinach and sprouts in the USA and German highlighted the public health concerns. It has been reported that at least 10 cells existing in food could cause human illness [6]. But Asai et al. [7] found that only 0.75 to 1.5 viable cells of *E. coli* O157:H7 in salted salmon roe could cause the infection. In order to explain this phenomenon, Makino et al. [8] re-estimated the number of *E. coli* O157:H7 cells in the implicated salted salmon roe from their membrane integrity, cell elongation and pathogenicity for mice, and found that almost all of *E. coli* O157:H7 cells had entered a viable but nonculturable (VBNC) state, thus the authors suggested that VBNC cells of *E. coli* O157:H7 in the food should be the source of the outbreak.

The VBNC state is a survival strategy adopted by numerous bacteria when exposed to harsh environmental stresses [9]. In this state, bacteria are unable to form colonies on conventional growth media, but are still viable and maintain metabolic activity [10]. But under appropriate conditions they can resuscitate to culturable state [11]. Therefore, pathogens in the VBNC state are able to escape conventional microbiological detection methods and thus pose a significant health risk. Up to now, a number of pathogens have been reported to be capable of entering the VBNC state, including *Campylobacter* spp., *E. coli*, *Legionella pneumophila*, *Listeria monocytogenes*, *Pseudomonas aeruginosa* and several *Salmonella* and *Vibrio* spp. [12]. The VBNC state of *E. coli* O157:H7 can be induced by a low temperature, starvation, exposure to chlorine and high osmotic pressure [8], [13], [14]. Resuscitation of *E. coli* O157:H7 cells from VBNC state has also been reported [8], [14], [15]. Furthermore, Liu et al. [16] confirmed the production of Shiga toxins (Stx) and the expression of both *stx*1 and *stx*2 toxin genes in VBNC *E. coli* O157:H7 cells. These results demonstrated that VBNC *E. coli* O157:H7 cells in food and water would cause a potential health risk.

Currently, thermal pasteurization techniques are widely used to prevent pathogens infection, but they also can damage some good properties of products, such as nutrition, color and taste. Therefore, non-thermal pasteurization techniques, including high hydrostatic pressure, pulsed high electric fields, high pressure CO_2_ (HPCD), irradiation and oscillating magnetic fields, have attracted much attention [17]. HPCD is one of the most promising techniques as it requires neither extreme pressure (below 50 MPa) nor extreme temperature (5–60°C). Various studies by using traditional culture methods have shown that HPCD is an effective means to inactivate microorganisms [18], [19], [20]. But in our previous study, we found that yeasts and moulds in apple juice subjected to HPCD treatment (20 MPa, 52°C, 30 min) could not be detected by conventional culture-based assay at initial storage time, but their counts increased to about 10 cfu/mL after 21-day storage at 28°C. These results suggested that VBNC cells might be induced by the HPCD treatment and then resuscitated during storage [21]. In addition, by using conventional cultivation-based technique and flow cytometer combining with PI/SYBR-Green I, Spilimbergo et al. also found that a significant portion of *Saccharomyces cerevisiae* cells injured by HPCD treatment at 36°C was incapable of forming colonies but was still integer and potentially metabolically active [22]. In this study, we investigated whether the VBNC state in *E. coli* O157:H7 can be induced by HPCD treatment, and its characteristics were further studied.

## Materials and Methods

### Bacterial Strain and Culture Conditions

*E. coli* O157:H7 NCTC 12900, a well characterized Stx negative strain, was obtained from the National Culture Type Collection (Colindale, London, United Kingdom). The strain was stocked in tryptic soy broth (TSB) with 25% glycerol at −80°C, and was activated by streaking onto TSA plate and incubating at 37°C for 24 h. And then, a single colony was picked and used to inoculate TSB and incubated at 37°C for 12 h with shaking at 200 rpm. This overnight culture was transferred to TSB at a dilution of 1∶100 and grew to the exponential phase (OD_550_ = 0.93). 10 mL of the exponential-phase cells were centrifuged at 8,000 g for 10 min at room temperature and pellets were washed twice with 0.85% (w/v) sterile NaCl solution (pH 7.0) and then resuspended in 20 mL of the same NaCl solution to a concentration of approximately 10^8^ cfu/mL. The inocula were freshly prepared according to the procedure described above in all of the experiments.

### VBNC State of *E. coli* O157:H7 Induced by HPCD Treatment

Induction of VBNC state by HPCD treatment was performed with a batch HPCD system without stirrer, which was designed by the China Agricultural University (Beijing, China) [19]. For each experiment, 20 mL of the cell suspensions was transferred to a 50 mL sterile glass tube and the tube was covered with a plastic film with a 0.22 µm membrane filter in the center for aeration and preventing microbial pollution. As the pressure vessel of the HPCD system reached the experimental temperature (25°C, 31°C, 34°C or 37°C), the sample tubes were placed in the pressure vessel. Then, the vessel was pressurized by the plunger pump to 5 MPa within 30 s. At 25°C, the treatment were respectively lasted for 10, 20, 30, 35 and 40 min. At 31°C, the treatment were respectively lasted for 10, 15, 20, 25 and 30 min. At 34°C, the experiments were respectively lasted for 6, 12, 18, 24 and 28 min. At 37°C, the experiments were respectively lasted for 5, 10, 15, 20 and 25 min. The carbon dioxide purity was 99.5% in all the experiment treatments. After HPCD treatment, the pressure vessel was depressurized within 30 s and the sample tubes were removed immediately. Then the tubes were shaken to remove dissolved carbon dioxide in samples and cooled at 4°C for further analysis. Each experiment was performed in duplicate.

### VBNC State of *E. coli* O157:H7 Determined by Enumeration Assays

Total cell counts and viable cell counts in the samples treated with HPCD were determined by staining with a Live/Dead *Bac*Light bacterial viability kits (Molecular Probes Inc., Eugene, OR, USA). This kit utilizes a mixture of two nucleic acid stains, SYTO 9 and propodium iodide (PI), to evaluate cell membrane integrity. SYTO 9 (which gives green fluorescence) can pass through the intact or damaged plasma membrane of bacterial cells, while PI (which gives red fluorescence) can penetrate only cells with damaged plasma membrane and can compete with SYTO 9 for nucleic acid binding sites. Therefore, the cells with intact cell membranes (considered to be alive) exhibit green fluorescence, whereas bacteria with damaged membranes (considered to be dead) exhibit red fluorescence. However, in some cases four color cells, green cells, yellow cells, orange cells and red cells, could be observed by fluorescence microscope. Therefore, flow cytometer was further used to determine the percentage of the green cells. After staining according to the manufacturer’s instructions, the cells were visualized under a D-eclipse C1 confocal laser scanning microscopy (CLSM) equipped with a Nikon TE 2000 U microscope (Nikon Corporation, Tokyo, Japan) for total cell counts and analyzed by a FACSCalibur flow cytometer (Becton Dickinson Immunocytometry Systems, San Jose, Calif. USA) for viable cell counts. In order to determine the culturability of the cells in the HPCD-treated samples, each sample was serially (1∶10) diluted with 0.85% NaCl solution and pour-plated on TSA in duplicate. When the culturable cells were less than 1 cfu/mL, 10 mL of the HPCD-treated cell suspensions were centrifuged and the pellets were resuspended in 1 mL of 0.85% NaCl solution and then pour-plated on TSA either supplemented with 0.2% sodium pyruvate (TSA-SP plates) or without supplement (TSA plates). When plate counts indicated a culturable cell concentration below 0.1 cfu/mL, the cells were considered to be in the nonculturable state [14], [23].

### Resuscitation Experiments of VBNC State of *E. coli* O157:H7

10 mL of the four HPCD-treated samples with less than 0.1 cfu/mL culturable cells were centrifuged at 8,000 g for 10 min, and the pellets were resuspended in the same volume of TSB, and then 10 mL of these cell suspensions and their 10-fold serial dilutions in TSB were transferred to 50 mL sterile glass tubes. The tubes were incubated at 37°C in a static state. All of the resuscitation samples were sampled at 6 h and 24 h to determine culturability by plating on TSA. The resuscitation experiment was carried out in duplicate.

### Analysis of Enzymatic Activity of VBNC *E. coli* O157:H7 Cells

To investigate the cellular enzymes of VBNC cells with less than 0.1 cfu/mL culturable cells induced by the HPCD treatments, the enzymatic activities of VBNC cells and exponential-phase cells were determined by an API ZYM kit (BioMérieux, Marcy l’Etoile, France). This system can monitor 19 different enzymatic activities from a complex sample. The VBNC cells and exponential-phase cells were respectively centrifuged at 8,000 g for 10 min and then resuspended in 0.85% NaCl solution to a density approximating McFarland no. 5 or 6 turbidity standard (Ref. 70900; BioMérieux, Marcy l’Etoile, France). The suspensions were added to the reaction strips and incubated at 37°C for 4 h in the dark, and then colorimetric reagents were added to the reaction system. After 5 to 10 min, each enzymatic activity was estimated according to the color.

### Resistance of VBNC *E. coli* O157:H7 Cells to Sonication

The procedure of sonication was performed as described previously with some modifications [24]. 20 mL of the exponential-phase cells and the VBNC cells with less than 0.1 cfu/mL culturable cells treated at 5 MPa and 25°C for 40 min was centrifuged at 8,000 g for 10 min at room temperature and resuspended in 4 mL of 0.85% NaCl solution, and then 2 mL of the concentrated sample was transferred to a 10 mL centrifuge tube and subjected to sonication on ice bath with a JY92-II sonicator (Ningbo Scientz Biotechnology Co. Ltd., Ningbo, China). Each sonication pulse lasted for 5 s with 5 s intervals, and the working power was 200 W. After treating for ten and twenty sonication pulses respectively, the sample was measured for the absorbance at 550 nm.

### Microscopic Observations of VBNC *E. coli* O157:H7 Cells

The scanning electron microscopy (SEM) and transmission electron microscopy (TEM) assays were used to observe the morphology and interior structure in *E. coli* O157:H7 cells. The exponential phase cells, the VBNC cells with less than 0.1 cfu/mL culturable cells (5 MPa, 25°C, 40 min) or 6 h and 24 h resuscitated cells from 10-fold dilution VBNC sample were rinsed in 0.85% NaCl solution (pH 6.8), and then centrifuged at 2700 g for 10 min at 4°C. The pellet was fixed initially in 3% (v/v) glutaraldehyde solution in 0.1 M phosphate buffer (PBS, pH 7.2) for 24 h. After the primary fixation, the cells in suspension were centrifuged, and then the pellet was post-fixed in 1% osmium tetroxide solution (pH 7.2). After 1.5 h, the cells were rinsed in PBS for three times, and were further dehydrated for 10 min in a series of cold ethanol solutions (10%, 30%, 50%, 70% and 90%), and then rinsed in 100% ethanol at 10 min intervals for two times. For SEM assay, the cells were rinsed twice with 100% isoamyl acetate for 15 min, critical point dried, and coated with gold palladium. Observation and photomicrographs were then carried out with a Hitachi S-3400 N SEM (Hitachi Instruments Inc., Japan). For TEM assay, the cells were infiltrated with the solution of acetone and epon-araldite over a period of 24 h, and polymerized at 60°C for 48 h. Fifty nanometer sections were sectioned, and then the sections were placed on copper sieves and contrasted with uranyl acetate and lead citrate for 30 min respectively. The sections were examined on JEM-1230 TEM (JEOL Japan Electronics Co., Ltd., Japan).

### Statistical Analysis

Analyses of variance (ANOVAs) were performed by using the software Microcal Origin 8.0 (Microcal Software, Inc., Northampton, MA, USA). The significance level was 0.05.

## Results

### Entry into the VBNC State

**Figure 1:**
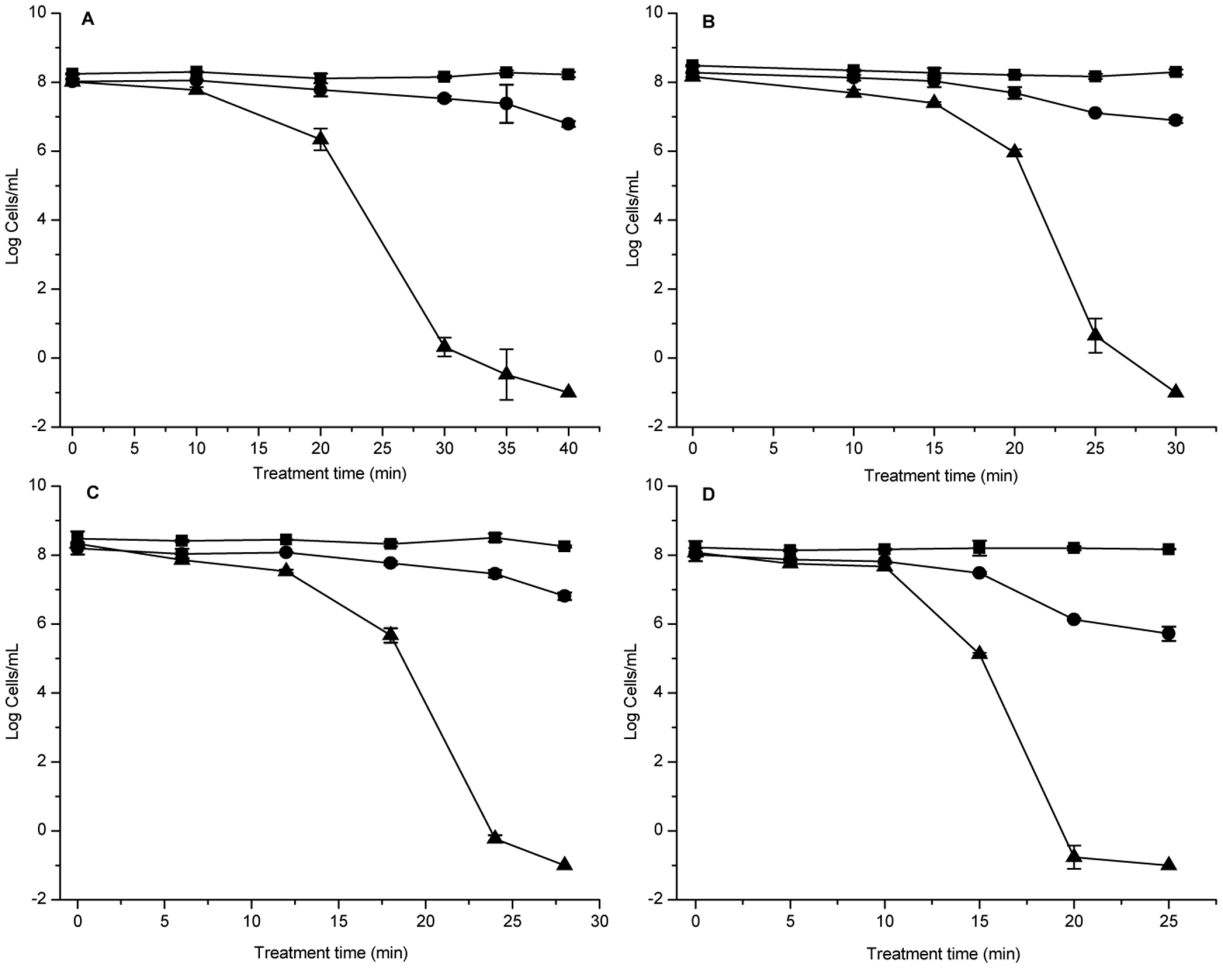
Entry of *Escherichia coli* O157:H7 into a VBNC state by HPCD treatment. (A) 5 MPa, 25°C; (B) 5 MPa, 31°C; (C) 5 MPa, 34°C; (D) 5 MPa, 37°C. Shown are plate counts (▴) on tryptic soy agar (TSA), and viable cell counts (•) and total cell counts (▪) by the Live/Dead staining method.

The changes in cell numbers of *E. coli* O157:H7 induced by HPCD treatment were investigated in this study. The results showed that the level of total cell counts always remained at about 10^8^ cells/mL at 5 MPa and 25°C, 31°C, 34°C or 37°C, while plate counts decreased to undetectable levels (<0.1 cfu/mL) at 40 min, 30 min, 28 min and 25 min, respectively (Fig. 1). However, viable cell counts still approximated 10^6^ cells/mL at the corresponding time point as shown in Fig. 1. These results indicated that all the four HPCD treatments in this study could induce the entry of *E. coli* O157:H7 cells into the VBNC state. But with the improvement of treatment temperature, the time of entry into VBNC state would shorten.

### Resuscitation Studies of VBNC Cells

**Figure 2:**
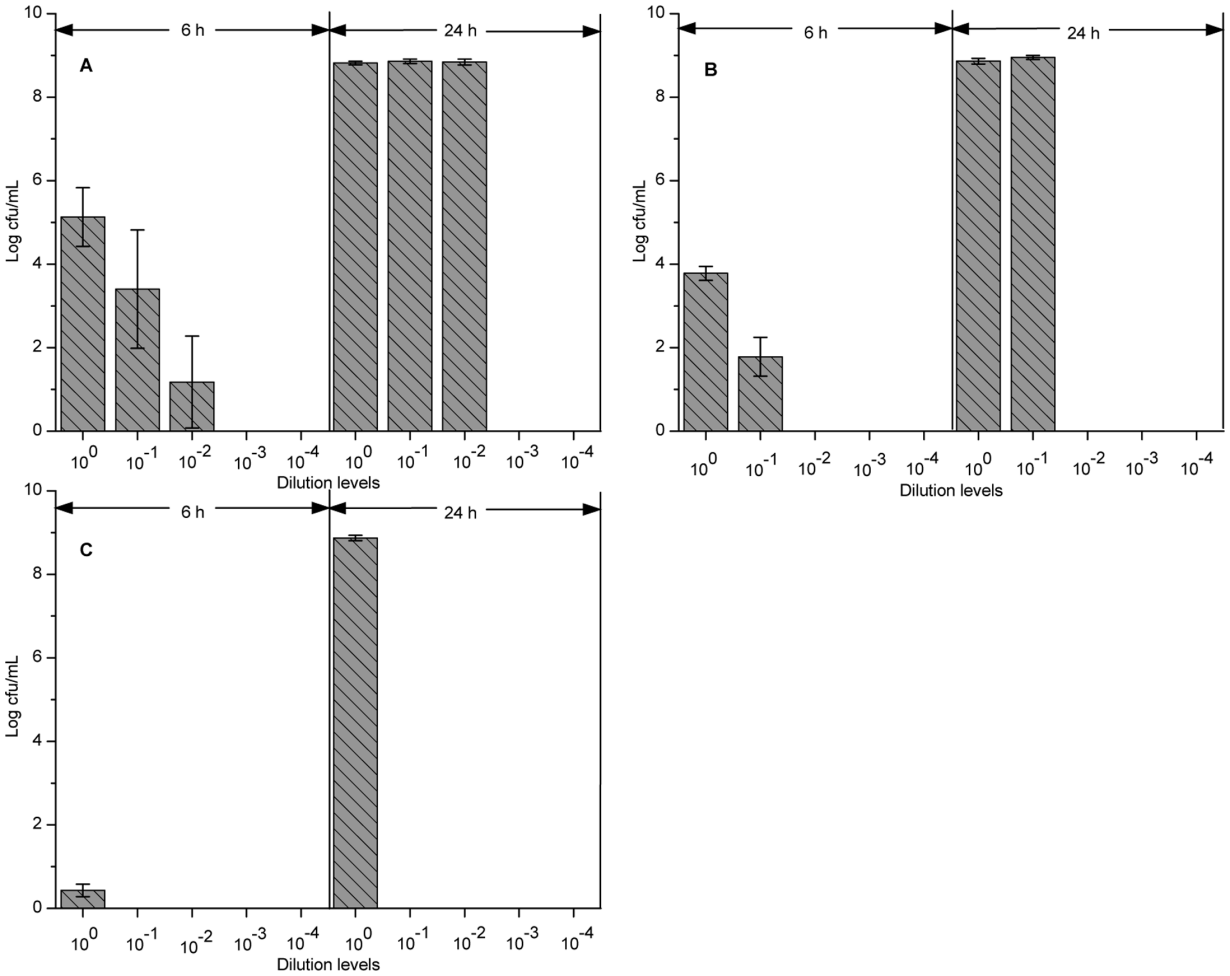
Resuscitation of VBNC *Escherichia coli* O157:H7 cells induced by HPCD treatment. (A) 5 MPa and 25°C for 40 min; (B) 5 MPa and 31°C for 30 min; (C) 5 MPa and 34°C for 28 min. Resuscitation samples were cultured in TSB and incubated at 37°C for 6 h and 24 h. Shown are plate counts for each 10-fold serial dilution (from 10^0^ to 10^−4^) sample.

To examine whether HPCD-induced VBNC cells with less than 0.1 cfu/mL culturable cells could resuscitate, VBNC cells with 10-fold serial dilutions were incubated in fresh TSB at 37°C for monitoring the culturable cells. Except of VBNC cells induced at 5 MPa and 37°C for 25 min, VBNC cells from other three HPCD treatments recovered culturability. When the resuscitation samples from VBNC cells treated at 5 MPa and 25°C for 40 min were incubated at 37°C for 6 h, the average plate counts of undiluted, 10^−1^- and 10^−2^-diluted samples were 1.34×10^5^, 2.54×10^3^ and 15 cfu/mL, respectively (Fig. 2A). This result indicated that resuscitation had occurred as the resuscitation sample with less than 1.0×10^−3^ cfu/mL culturable cells also restored culturability. When the incubation time was prolonged to 24 h, the culturable cell counts of these three resuscitation samples all rose to about 6.89×10^8^ cfu/mL (Fig. 2A). This result indicated that the cells after 24 h resuscitation were in the stationary phase which was caused by the multiplication of the resuscitated cells. Resuscitation of VBNC cells treated by HPCD at 31°C or 34°C also showed the same phenomenon (Fig. 2B or C). However, only 10^−1^ and undiluted samples could resuscitate at 31°C and only undiluted samples could resuscitate at 34°C (Fig. 2B or C). These results suggested that, with the improvement of HPCD treatment temperature, resuscitation capability of VBNC cells decreased.

### Enzymatic Activity of VBNC Cells

**Figure 3:**
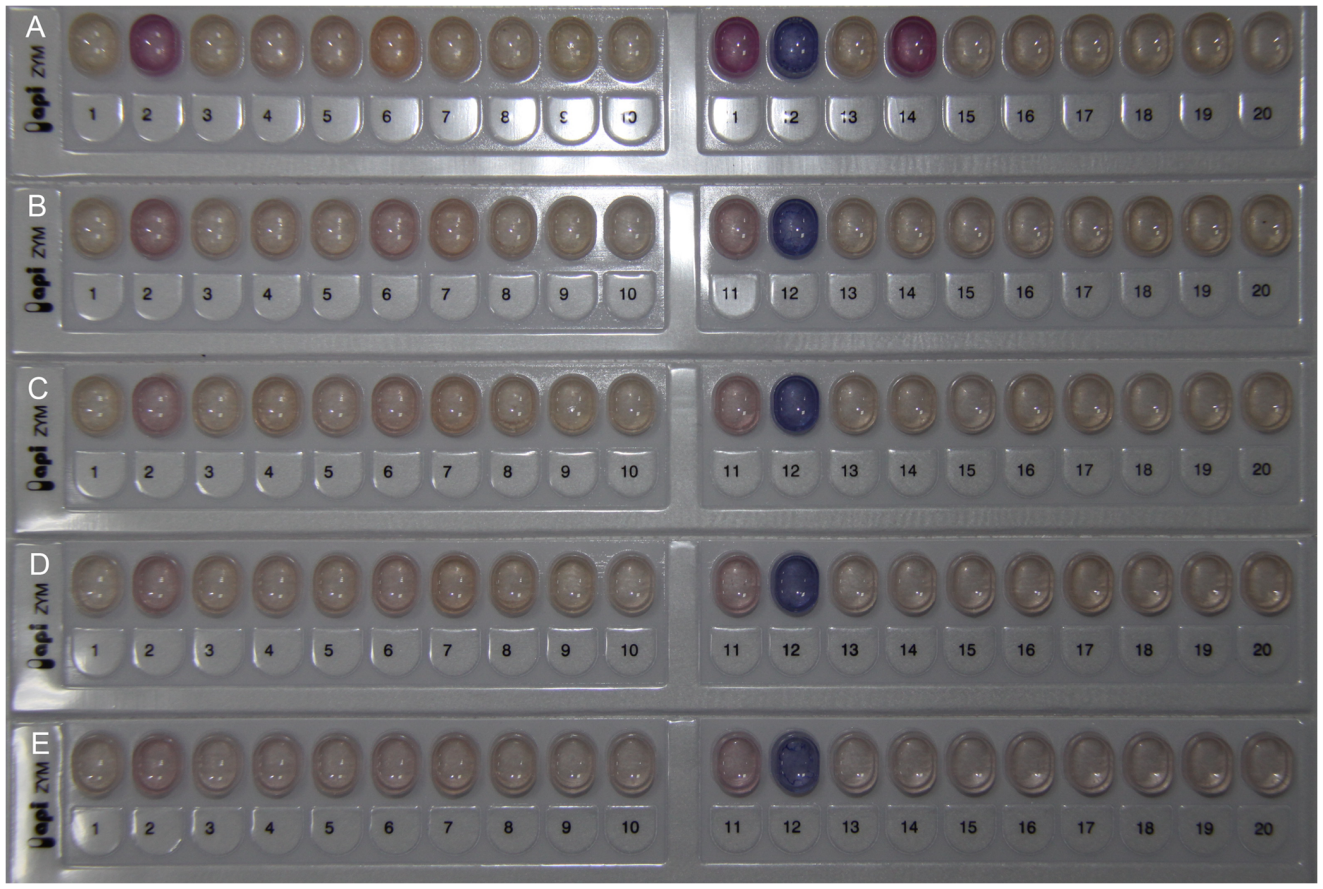
Enzymatic activities of *Escherichia coli* O157:H7 measured by the API ZYM system. (A) the exponential-phase cells; (B) VBNC cells treated by HPCD at 5 MPa and 25°C for 40 min; (C) VBNC cells treated by HPCD at 5 MPa and 31°C for 30 min; (D) VBNC cells treated by HPCD at 5 MPa and 34°C for 28 min; (E) VBNC cells treated by HPCD at 5 MPa and 37°C for 25 min.

Because enzymes are essential to metabolic processes in microorganisms, the cellular enzyme activities in HPCD-induced VBNC cells with less than 0.1 cfu/mL culturable cells were investigated in this study. As shown in Fig. 3, alkaline phosphatase (No. 2), leucine arylamidas (No. 6), acid phosphatase (No. 11), naphthol-AS-BI-phosphohydrolase (No. 12) and β-galactosidase (No. 14) were identified in the exponential-phase cells. However, only alkaline phosphatase, acid phosphatase and naphthol-AS-BI-phosphohydrolase were identified in HPCD-induced VBNC cells, and activities of these enzymes were lower than those in the exponential-phase cells (Fig. 3). According to the color intensity of each enzyme, activities of the enzymes in HPCD-induced VBNC cells decreased with increasing the HPCD treatment temperature (Fig. 3). These results suggested that HPCD-induced VBNC cells still retain metabolic activity, but it was lower than the exponential-phase cells. Such reduction of enzymatic activity led to a low level of metabolic activity in VBNC cells, which could be a survival strategy that allows the VBNC cells to minimize cellular energetic requirements.

### Mechanical Resistance of VBNC Cells to Sonication

**Figure 4:**
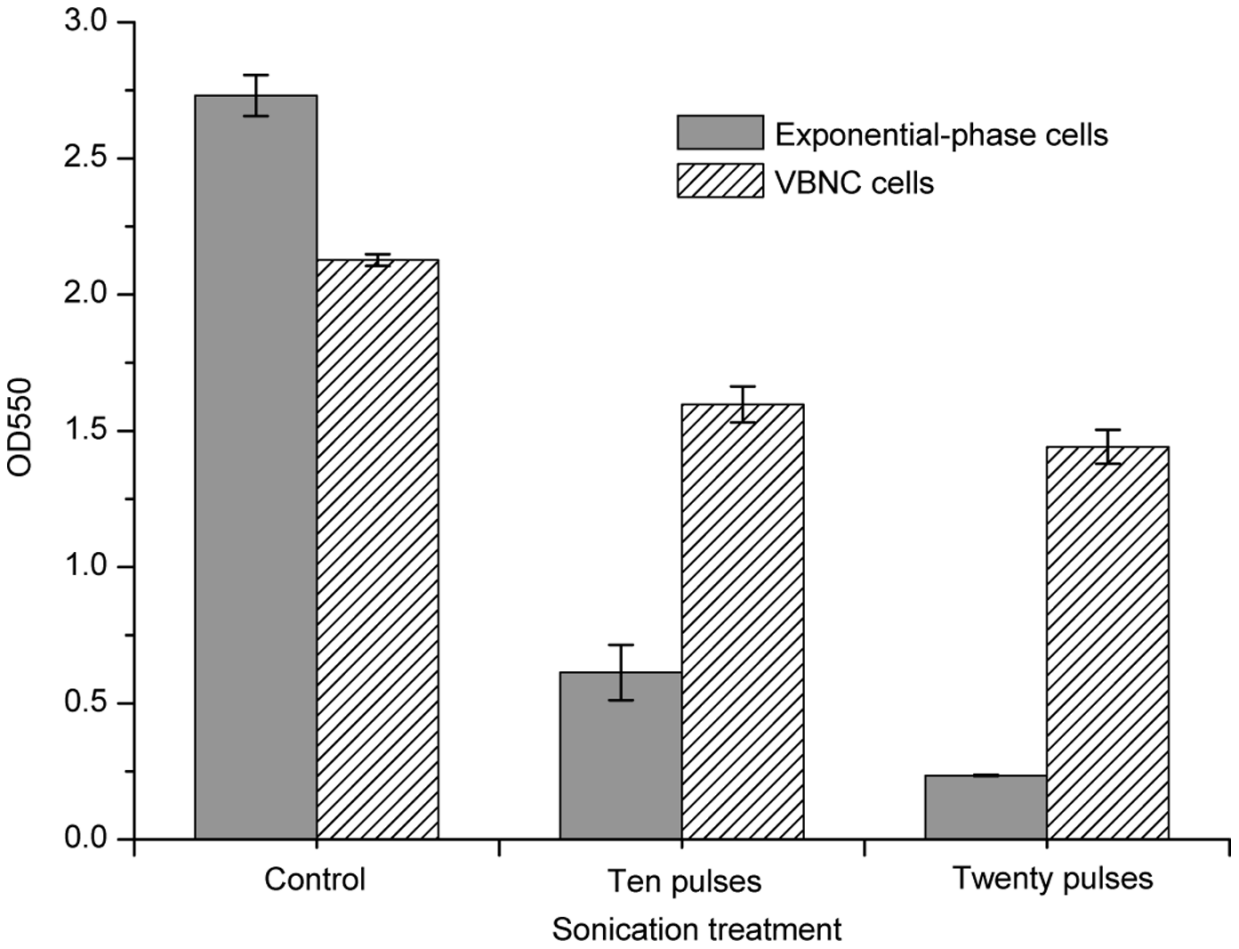
Resistance of VBNC *Escherichia coli* O157:H7 cells to sonication. The VBNC *E. coli* O157:H7 cells were induced by HPCD treatment at 5 MPa and 25°C for 40 min.

In order to determine the mechanical resistance of VBNC cells, the susceptibility of the exponential-phase cells and VBNC cells treated at 5 MPa and 25°C for 40 min to sonication was compared. The results showed that absorbance of the VBNC cells at 550 nm decreased to 75% of the initial value (OD_550_ = 2.13) after sonication for ten pulses, while only about 22% of the initial absorbance value (OD_550_ = 2.73) was detected for the exponential-phase cells (Fig. 4). When the sonication treatment was prolonged to twenty pulses, absorbance of the VBNC cells remained almost constant, while absorbance of the exponential-phase cells at 550 nm decreased to 0.24 (Fig. 4). These results suggested that HPCD-induced VBNC cells of *E. coli* O157:H7 displayed a higher resistance to sonication than the exponential-phase cells.

### Electron Microscopy

**Figure 5:**
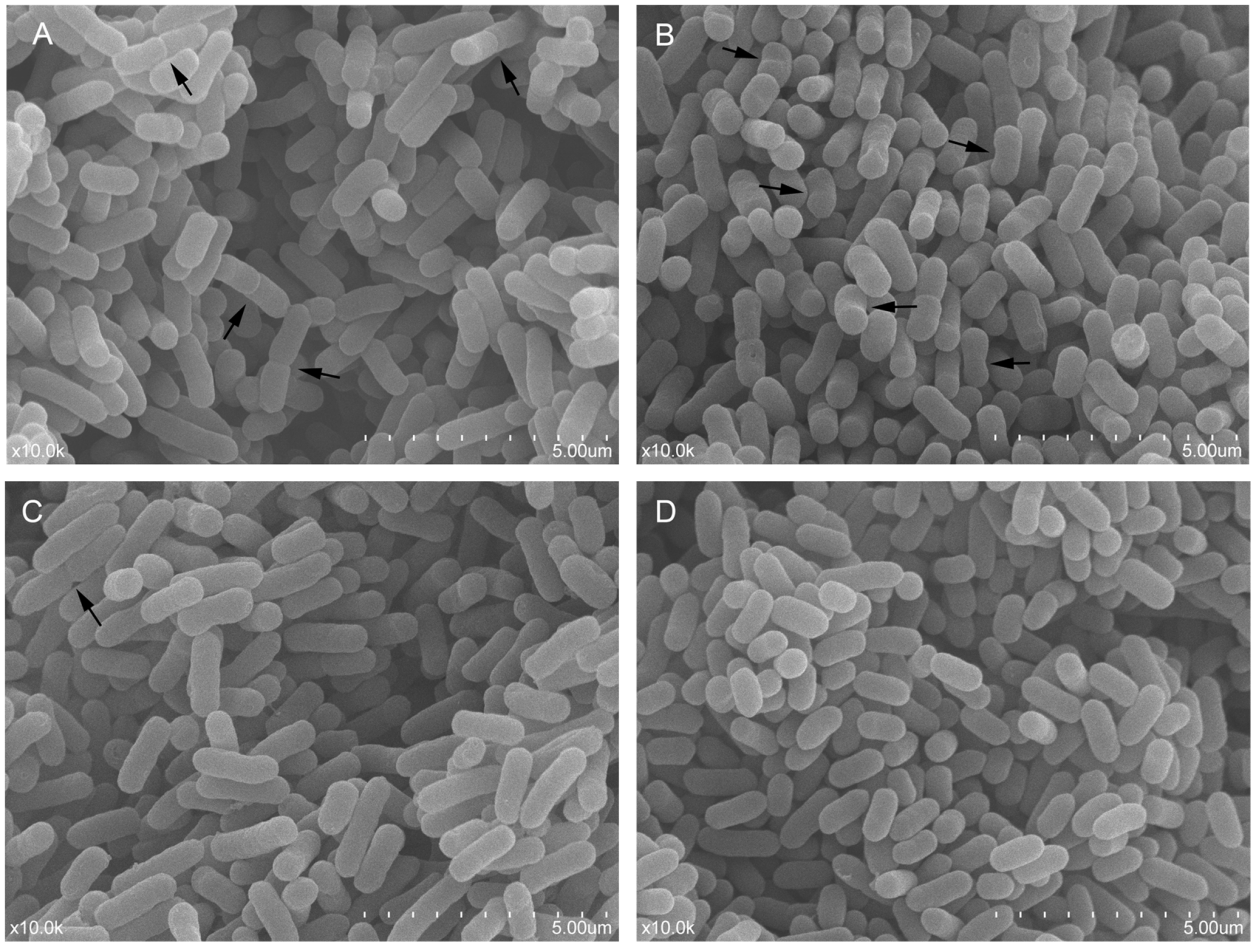
Scanning electron micrograph (magnification of × 10,000) of *Escherichia coli* O157:H7. (A) the exponential-phase cells; (B) VBNC cells induced by HPCD treatment at 5 MPa and 25°C for 40 min; (C) resuscitated cells cultured in TSB for 6 h; (D) resuscitated cells cultured in TSB for 24 h.

The morphology of the exponential-phase cells of *E. coli* O157:H7 were rod-shaped with a smooth surface, and the size of the cells was relatively uniform, averaging 1.54 µm in length. Meanwhile, cell division by binary fission was also observed by SEM (Fig. 5A, arrow). In the VBNC state, cells were mostly curved rods (Fig. 5B, arrow) with 1.45±0.11 µm long, and the cell surface was relatively rough (Fig. 5B). Upon 6 h of resuscitation, most of the cells changed from curved rods to long rods, and the length of the cells was 1.97±0.50 µm, which was longer than the exponential-phase cells (Fig. 5C). Meanwhile, fewer binary fission cells were present in the 6 h resuscitated cells (Fig. 5C, arrow), which indicated that some resuscitated cells had already multiplied. When the resuscitation time prolonged to 24 h, size of the rod-shaped cells decreased, with length only three-quarter of the exponential-phase cells, averaging 1.10 µm (Fig. 5D), which indicated that the cells were in the stationary phase.

**Figure 6:**
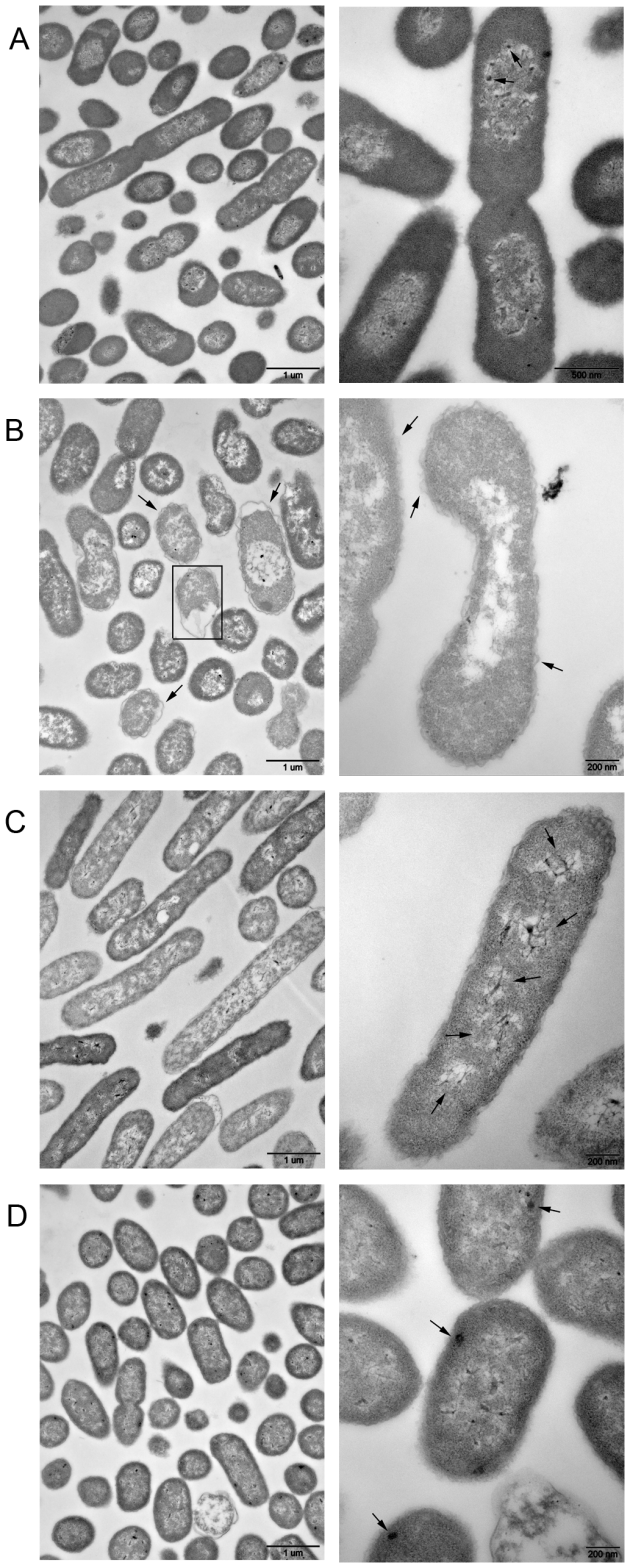
Transmission electron micrograph of *Escherichia coli* O157:H7. (A) the exponential-phase cells; (B) VBNC cells induced by HPCD treatment at 5 MPa and 25°C for 40 min; (C) resuscitated cells cultured in TSB for 6 h; (D) resuscitated cells cultured in TSB for 24 h. Left images, magnification of × 25,000; right images, except in panel A, magnification of × 80,000; right image of panel A, magnification of × 60,000.

The interior structure of the exponential-phase cells, the VBNC cells and the resuscitated cells was observed by TEM. In the exponential-phase cells, nucleoid (low electronic density area) was in the center of the cells, and cytoplasmic matrix (high electronic density area) filled with ribosomes was around the nucleoid (Fig. 6A). In the nuclear region, several dark granules (Fig. 6A, right image, arrow) were combined with nucleic acid material. This combination would promote the cell growth and multiplication of the exponential-phase cells. However, cytoplasmic matrix in the VBNC cells (Fig. 6B) was much less dense than that of the exponential-phase cells (Fig. 6A), which was caused by a reduction in the number of ribosomes; nucleoid in the VBNC cells was also less dense, and nucleic acid material was loosened (Fig. 6B). There was no combination of dark granules and nucleic acid material in the nucleoid of the VBNC cells (Fig. 6B). Additionally, a gap was formed between the outer and inner membranes in the VBNC cells (Fig. 6B, arrow). Some cells contained less internally staining materials which are believed to be dead (Fig. 6B, left image, rectangle). When the VBNC cells were resuscitated for 6 h, the cytoplasm of the resuscitated cells (Fig. 6C) was more densely stained than that in the VBNC cells (Fig. 6B), and more than three nucleoids distributed throughout the cytoplasm (Fig. 6C, right image, arrow). More nucleoids could promote the accumulation of cell components within a short time, which would accelerate the cell growth and multiplication. After 24 h of resuscitation, the central part of the shortened rod cells was densely stained (Fig. 6D) and some densely stained granules were located exclusively in the peripheral part of the cytoplasm (Fig. 6D, right image, arrow). These densely stained granules (Fig. 6D) were probably polyphosphate particles which are found in some bacteria under starvation stress [25].

## Discussion

In our preliminary study, *E. coli* O157:H7 cells with intact membrane (probably be VBNC cells) were also found when they were treated under HPCD conditions above the critical point of CO_2_, but the proportion of these cells with intact membrane was lower than that treated under mild HPCD conditions below the critical point. As more VBNC *E. coli* O157:H7 cells were needed for investigating their characteristics, we chose the mild HPCD conditions in this study to induce the VBNC state in *E. coli* O157:H7 cells. In this study, we reported for the first time that HPCD treatment could induce *E. coli* O157:H7 in 0.85% NaCl solution (pH 7.0) to enter the VBNC state. During HPCD treatment, bacterial cells would suffer from stresses of high pressure, lowered pH caused by dissolved CO_2_ and high CO_2_ concentration. These stresses might synergistically induce the VBNC entry. In the induction study, we found that the time required for *E. coli* O157:H7 to become nonculturable was shortened as the HPCD treatment temperature increased. Similar observations were reported by Rollins and Colwell [26] and Wong and Wang [27] for *C. jejuni* and *V. parahaemolyticus* in nutrient-limited microcosms, respectively. They all found that the loss of culturability of cells was accelerated at higher temperatures (37°C versus 4 or 15°C). During HPCD treatment, higher temperature can stimulate the diffusivity of CO_2_ and increase the fluidity of the cell membrane [17], which will enhance the interaction between CO_2_ and cells, thus might accelerate the loss of culturability. Besides temperature, pressure is another main parameter during HPCD treatment, which can increase the solvating power of CO_2_ [17]. Although the effect of pressure on the entry of *E. coli* O157:H7 into the VBNC state was not tested, higher pressures during HPCD treatment could accelerate the VBNC entry.

Morphological and interior characteristics of VBNC cells have been studied by some researchers [26], [28], [29], [30]. When entering the VBNC state, some bacteria showed a reduction in size, such as *E. coli* [24] and *V. cincinnatiensis* [30], while other bacteria showed a slightly bigger in cell size, such as *Enterococcus faecalis* [31] and *Erwinia amylovora* [32]. Additionally, few of studies found a non-detectable or a small change in cell size in VBNC cells [33], which correlated with the findings in this study. Although there was a small change in cell size, the morphology of the VBNC *E. coli* O157:H7 cells changed into a curved rod shape with relatively rough surface (Fig. 5B). Furthermore, compared with the exponential-phase cells, the nucleic acid material was loosened without “dark granules” binding and the number of ribosomes was obviously reduced in the VBNC cells. These changes in the interior structure possibly prevent DNA amplification and protein translation, thus reduce macromolecular synthesis and nutrient transport in VBNC cells. Overall, changes in morphology and interior structure might well be cellular strategies that allow VBNC cells to minimize cell maintenance requirements [10] and eventually enhance cell survival under adverse conditions. Therefore, the resistance of the VBNC *E. coli* O157:H7 cell to sonication was further analyzed in this study.

Up to now, several studies have found that VBNC cells exhibited an increased resistance to various stresses, such as thermal treatment, low salinity, ethanol treatment, acidic stress and sonication [27], [34], [35]. As expected, the VBNC cells of *E. coli* O157:H7 induced by HPCD treatment showed greater resistance to sonication than the exponential-phase cells. Weichart and Kjelleberg [36] suggested that the resistance of VBNC *V. vulnificus* cells to stress conditions might partly be correlated with modulations in the cell wall composition. Signoretto et al. [31] also ascribed the increased resistance of VBNC *E. faecalis* cells to mechanical disruption to the higher cross-linking in cell wall peptidoglycan of the VBNC cells. With respect to *E. coli*, an increase in cross-linking of peptidoglycan was also found in the VBNC cells when compared with culturable cells [24]. Therefore, the mechanical resistance of the VBNC cells to sonication in this study might partly be caused by the modifications in the cell wall composition. On the other hand, when entering the VBNC state, the number of ribosomes was decreased, nucleic acid material was loosened, and a gap was formed between the outer and inner membranes. These changes make the VBNC cells more resilient and thus make the VBNC cells more resistant to mechanical stress than the exponential-phase cells.

Although difference between the number of active cells and culturable cells is usually used as an indication of the existence of the VBNC state in bacteria, resuscitation also serves as a definitive confirmation that nonculturable cells were indeed VBNC. However, some researchers ascribed the phenomenon of resuscitation to regrowth of an undetectable level of culturable cells [37], [38], and Bogosian et al. [37] is the main supporter for this opinion, who proposed that resuscitation of cold-shocked cells of *V. vulnificus* was owing to the growth of the residual hydrogen peroxide-sensitive culturable cells. But various approaches were taken to examine whether true resuscitation of VBNC cells occurs, such as comparison study for resuscitation rate and growth rate [39], [40], dilution experiment [32], [41], and addition of hydrogen peroxide-removers (e.g. catalase or SP) [39] or antibiotics [42]. In this study, in order to differentiate resuscitation from regrowth, dilution method was carried out; meanwhile, we ruled out the possibility that resuscitation is due to the regrowth of hydrogen peroxide-sensitive culturable cells by detecting the VBNC cells using TSA-SP. Moreover, the difference of morphological and interior characteristics between 6 h resuscitated cells (Fig. 5C and 6C) and exponential-phase cells (Fig. 5A and 6A) also suggested that the resuscitation was a unique process and not caused by the cell regrowth. In addition, although resuscitation failed for VBNC cells induced by HPCD treatment at 37°C in this study, it did not mean that the VBNC cells induced at this condition were not able to resuscitate. Perhaps the resuscitation method described here was not the optimal condition for restoring the culturability of the HPCD-induced VBNC cells. Taken together, results presented here suggested that resuscitation really occurred in the VBNC *E. coli* O157:H7 cells induced by HPCD treatment. Therefore, in order to effectively kill HPCD-induced VBNC *E. coli* O157:H7 cells, the formation mechanisms of the VBNC cells under HPCD treatment should be elucidated in future work. Furthermore, as some genes still can transcribe in VBNC state of microorganisms [43], [44], it is feasible to detect if there exist VBNC cells in HPCD-treated products by molecular-based methods [45] for food safety.
